# ctDNA and CTCs in Liquid Biopsy – Current Status and Where We Need to Progress

**DOI:** 10.1016/j.csbj.2018.05.002

**Published:** 2018-06-01

**Authors:** Martin H.D. Neumann, Sebastian Bender, Thomas Krahn, Thomas Schlange

**Affiliations:** a Bayer AG, Pharmaceutical Division, Biomarker Research, Wuppertal, Germany; b Bayer AG, Pharmaceutical Division, Translational Assay Technology, Berlin, Germany

**Keywords:** Liquid biopsy, Circulating tumor DNA, Circulating tumor cells, Pre-analytical handling, Technology overview

## Abstract

We discuss the current status of liquid biopsy and its advantages and challenges with a focus on pre-analytical sample handling, technologies and workflows. The potential of circulating tumor cells and circulating tumor DNA is pointed out and an overview of corresponding technologies is given.

## 1 Liquid Biopsy

### 1.1 Definition and Concept of Liquid Biopsy

Almost 150 years ago in 1869, the pathologist Thomas Ashworth provided evidence for the presence of circulating tumor cells (CTCs) in the blood of a metastatic cancer patient and, therefore, described for the first time a phenomenon nowadays considered as liquid biopsy. Different analytes can be identified in patient blood samples: circulating rare cells (such as CTCs, progenitor and mature endothelial cells or tumor-educated platelets), circulating cell-free DNA (ccfDNA, especially circulating tumor DNA, ctDNA), circulating cell-free RNA (ccfRNA) or extracellular vesicles (exosomes) and their cargo (including nucleic acids and proteins) [1]. Other blood components recently introduced as blood-based biomarker sources, like exosomes or platelets, but are beyond the scope of this review which will focus on ctDNA and CTCs. All these analytes can be used to increase our knowledge about the underlying disease (e.g. tumor burden and heterogeneity) ultimately translating into improved cancer diagnosis, therapy guidance and disease surveillance. To do so, several biological and technological challenges need to be overcome. In particular, standardized pre-analytical sample handling procedures as well as not yet available robust and reproducible workflows for the molecular analysis of liquid biopsy samples are desperately needed.

The concept of liquid biopsy aims at simple, fast and cost efficient monitoring of disease status or response to treatment. Here, liquid biopsy offers several advantages compared to “conventional” tissue biopsy: Liquid biopsy is less burdensome than a tissue biopsy, because body fluids like blood, saliva or urine are much easier to access. For some diseases such as lung cancer taking a tissue biopsy is clinically often not possible, e.g. due to a high risk of bleeding, nerve injury or disease spreading [2]. Moreover, tissue biopsies may not appropriately reflect the complex molecular profile of a primary tumor, because of its intratumoral or spatial heterogeneity, which can only be addressed by taking biopsies from different tumor areas [3]. Compared to this, liquid biopsies may offer a more comprehensive cross-section of heterogeneous diseases [4]. Furthermore, liquid biopsies may also provide insights into the molecular drivers of different primary tumors or metastases, which may significantly differ in the same patient. Since the genome of tumor cells is often highly unstable and susceptible to changes under different selective pressures (e.g. therapy) liquid biopsy may allow longitudinal disease surveillance to monitor developing tumor heterogeneity [5]. Overall, the liquid biopsy concept complements the personalized medicine approach and provides an innovative way for patient selection in clinical trials, here mutational analysis supports patient eligibility for targeted therapy [6, 7].

### 1.2 Requirements for Liquid Biopsy Technologies in the Clinical Setting

#### 1.2.1 Importance of Pre-Analytical Sample Handling

Most therapy decisions in the clinic are based on laboratory tests. This testing process can be divided into three phases: the pre-analytical phase, the analytical and the post-analytical phase [8, 9]. While the pre-analytical phase includes the identification and selection of an appropriate test, specimen collection and transport, the analytical phase comprises mainly the laboratory testing itself. The post-analytical phase consists of, for example, data analysis, interpretation of results and reporting, but also - if applicable - archiving of the remaining material. In the whole testing process, 46% to 68% of errors occur in the pre-analytical phase, which adversely influence the quality of the data in the following phases, leading to an increase of diagnostic costs and suboptimal or even wrong treatment decisions for the patient [10]. The most common mistakes include the selection of inappropriate tests, the use of inappropriate blood collection tube (BCT), poor sample collection procedures (e.g. hemolysis or insufficient volume) or wrong sample storage and transportation but also inaccurate sorting, aliquoting or technical mistakes (e.g. pipetting or centrifugation [8]). As a result, about 10% of patient deaths and 17% of adverse events are reported to be caused by such pre-analytic mistakes [11]. Due to the high relevance of preanalytical sample handling, significant efforts have been made to standardize processing and analysis of blood samples for different technologies. For example, van Ginkel and colleagues assessed a technical workflow to use highly sensitive Droplet Digital PCR (ddPCR) to detect rare mutational targets by analyzing ctDNA. They used blood samples from healthy donors and lung cancer patients to compare methods, protocols of sample collection, storage, centrifugation, isolation and quantification. They received, for example, highest cfDNA concentrations from serum tubes with most consistent results by using the QIAamp Circulating Nucleic Acid Kit [12]. Others performed comparative studies on extraction methods and analysis platforms, also for other liquid biopsy biomarkers like miRNAs [13]. The results of these studies corroborate that the outcome of an assay is highly dependent on multiple factors during the preanalytical sample workflow, which has to be taken into account for effective implementation of an assay in clinical practice.

This underlines the need to optimize pre-analytical handling also in the field of liquid biopsy. Therefore, various efforts are currently being made to establish best practice in the field, including the European FP7 consortium SPIDIA4P (Standardization and improvement of generic Pre-analytical tools and procedures for In-vitro DIAgnostics, http://www.spidia.eu/). Among the aims of SPIDIA4P are the stabilization, handling and study of biomolecules in liquid biopsies and tissues.

#### 1.2.2 Need for Standardization of Integrated Analytical Workflows

Regarding clinical utility of liquid biopsy as well as usefulness for research, it is very important to have easy-to-use, robust and reproducible workflows. Currently, there are no integrated, multicenter-tested workflows available covering the requirements for the clinical setting. Such workflows should include Standardized Operating Procedures (SOPs) for all above mentioned phases of laboratory testing starting with specimen collection and ending with result interpretation e.g. via bioinformatics analysis. Blood collection should be performed using certified BCTs, suitable for the respective downstream application (e.g. use of compatible stabilizer or fixative, volume adjusted). Next, fixed established protocols for specimen handling, storage and shipping of the blood sample or how to generate blood plasma (e.g. centrifugation) are indispensable. Depending on the analyte (CTCs, ctDNA, miRNA, etc.), robust extraction, isolation and quantification methods, are necessary. The implementation of an optimized preparation method for each analyte at the analytical sites has to be validated and documented for evaluation of the results. Multicenter comparisons of downstream read-out technologies (e.g. massive parallel sequencing for ctDNA or fluorescence-based quantification for rare cells) are also of key importance. Although many different liquid biopsy technologies appeared on the market in recent years [14], there is still a lack of technologies offering reproducible, robust, cost-effective and easy-to-use workflows from the sample to clinically meaningful data. To this day, there is only one FDA approved CTC quantification technology available for three metastatic tumor indications: the CellSearch system (Menarini Silicon Biosystems, Inc. [15]). For the detection of *EGFR* mutations in NSCLC patients a ctDNA based test received approval by the FDA in 2016 (https://www.fda.gov/Drugs/InformationOnDrugs/ApprovedDrugs/ucm504540.htm).

Public private partnerships have been initiated focusing on standardization of methods and technologies for the analysis of circulating nucleic acids and rare cells. The Innovative Medicines Initiative (IMI) program CANCER-ID (www.cancer-id.eu) is supported by financial contributions from the European Union‘s Seventh Framework Programme (FP7/2007-2013) and EFPIA (European Federation of Pharmaceutical Industries and Associations) companies’ in-kind contributions. Key deliverables of the consortium are the evaluation of technologies for blood-based biomarker analysis and establishment of criteria for benchmarking different technologies. This includes best practice recommendations for sample collection, protocols for specimen storage and shipment allowing bio- banking, as well as comparative data on different methods for the molecular analysis of CTCs, ctDNA and miRNAs. Ultimately, CANCER-ID aims to provide a basis for the use of blood-based biomarkers in multi-centered clinical trials. Another public-private partnership, the US based Blood Profiling Atlas in Cancer (BloodPAC) consortium (https://www.bloodpac.org/), has recently been established with the goal to support the development of liquid biopsy technologies in cancer research. BloodPAC is systematically collecting and harmonizing data from public and private research efforts utilizing CTC, ctDNA and some other analyte classes such as exosomes. This data is subsequently shared between the different stakeholders to identify best practice procedures.

The comprehensive evaluation of several different technologies as well as integration of these technologies in multicenter clinical studies is a tremendous effort, which is not easily feasible for a single pharmaceutical or academic institution. Programs such as CANCER-ID aim to accelerate the development of emerging technologies by defining end-user requirements. The demand for readily accessible (i.e. longitudinal) predictive data to support patient selection, to detect early signs of efficacy or to monitor the development of resistance towards targeted therapies in phase 2 and 3 clinical studies, a promise of liquid biopsy, makes such consortia attractive for the pharmaceutical and diagnostic industries.

Finally, world-wide availability of technologies and the regulatory approval of liquid biopsy devices and technologies have to be ensured.

In the following chapters an overview of some more widely used technologies to analyze liquid biopsy analytes such as rare cells or circulating nucleic acids will be provided.

## 2 Classes of Blood-Based Biomarkers in Liquid Biopsy

### 2.1 Rare Cells

Rare cells are generally considered low abundant cells in the blood stream, typically with a concentration below 1 in 10^5^ cells. Circulating mature endothelial cells (CECs), which are potential biomarkers for endothelial dysfunction in cancer, diabetes, cardio-vascular or acute kidney diseases [[16], [17], [18]] have been observed with a frequency of 10–100 CECs in 10^6^–10^8^ white blood cells, depending on the method of enrichment and detection. Compared to that, the estimated frequency of CTCs is even lower, ranging from 1 to 10 CTCs in 10^6^–10^8^ white blood cells. Therefore, the detection, quantification and isolation of single cells are challenging.

The potential of rare cells as blood-based biomarkers, especially CTCs, is underlined by many publications showing their clinical relevance [19]. CTCs are considered to be the main source of metastases [20]. Moreover, the number of CTCs in the blood correlates with reduced progression-free and overall survival [21, 22] and is of higher prognostic value than conventional imaging [23]. A big hurdle for further downstream analysis of CTCs is an efficient and reliable method for isolating these cells. Several technologies have been developed to separate rare cells in the blood from the extraordinarily high background of normal blood cells, mainly erythrocytes and leukocytes. Enrichment, separation or quantification of rare cells can be done either by physical properties like cell size, density or deformability and/or biological properties of the cells (e.g. marker gene expression on the cell surface). The size of CTCs ranges from 4 μm to 50 μm [24]. Furthermore, CTCs are described to be more rigid than hematopoietic cells [25, 26]. Detection and enrichment of CTCs based on their biological properties such as surface marker expression strongly depends on the availability of the respective antibody. This is further complicated by the potentially dynamic nature of marker expression, best exemplified in the case of CTCs undergoing Epithelial-to-Mesenchymal Transition (EMT) [27].

### 2.2 Principles of Rare Cell Enrichment and Detection

Gradient-based centrifugation (e.g. using a Ficoll gradient) is the easiest method to enrich for a specific cell type with a certain density. However, this result in an erythrocyte-depleted peripheral blood mononuclear cell fraction that requires further processing (e.g. cytospins) to allow staining for CTC marker expression. More recently, improved devices have been developed that integrate and automate some of these processing steps, e.g. the Rarecyte system [28].

Filtration enables enrichment of rare cells based on cell size. Examples are VyCAP-or ISET-filtration [29, 30] [31], which has been used successfully to isolate CTCs from lung and prostate cancer in clinical studies. In comparison with the only FDA cleared CTC quantification system – CellSearch – a higher CTC positivity in lung cancer (80% vs. 23%) [32] and prostate cancer (100% vs. 90%) [33] was shown. Since the CellSearch system enumerates cells that express the epithelial marker EpCAM, these differences suggest that there are additional CTC subpopulations or CTC-like cell types lacking EPCAM expression. Based on recovery rates using tumor cell lines spiked into blood Coumans and colleagues [25] suggest the use of filter material made out of a stiff, flat material, which is biocompatible and does not interact with any blood components e.g. triggering clotting.

Another CTC enrichment principle is using microfluidics, which allows separation of CTCs from other blood components at a constant flow. In comparison to filtration methods, microfluidic systems allow to harvest a CTC-enriched cell suspension for downstream analysis such as immunofluorescent labelling for single cell isolation. This is usually not readily achieved in a gentle and destruction-free manner with filtration systems due to the relatively tight binding of the cells to the filtration matrix during the pressurized filtration process. To overcome this, some technology providers are developing solutions to punch out the cells from well-shaped filter pores (e.g. VyCAP filters [34]). Further examples for more widely used microfluidic devices are the Parsortix device [35, 36], the ClearCell® FX1 [37] or the Vortex trapping system [38, 39].

CTCs and possibly other rare cells can also be separated based on differences in their electrical charge. Here, levitation due to di-electrophoretic (DEP) forces as well as hydrodynamic lift forces are applied to a cell suspension which is situated at a constant flow. This removes the vast majority of white blood cells (WBCs) to enrich the tumor cell fraction. This principle of combining density and electric charge is realized by e.g. ApoStream system [40].

For the enrichment of CTCs immunomagnetic methods have been widely applied. The AdnaGen test enables enrichment of CTCs using antibody-coated magnetic particles to enrich for tumor cells expressing specific cell surface markers including markers for EMT. This enrichment is followed by RT-qPCR analysis and subsequent determination of CTC numbers by Ct-values (e.g. via cytokeratin expression). The FDA-approved CellSearch® system for CTC enumeration is based on immunomagnetic EpCAM-dependend enrichment of CTCs. Well trained staff is required to evaluate the suggested CTCs based on single channel and overlay images of all three analyzed markers: CD45 (negative selection of leukocytes), EpCAM and Cytokeratin (both for CTC positive selection). Furthermore, an additional marker can be intergration in the CellSearch System such as for example HER2 [41] which allows differentiation of CTC subpopulations of breast cancer (e.g. EpCAM+/HER2− vs. EpCAM+/HER2+). However, all immunomagnetic enrichment methodologies do not enable CTC single cell isolation, which would be needed to increase our knowledge about intratumoral heterogeneity. Some commercially available systems such as a modified VyCAP filter (combined with a puncher device, see above) and most notably the DEParray system (Menarini Silicon Biosystems, Inc) are capable of CTC single cell isolation. At the research level micromanipulation (e.g. CellCelector) and single cell sorting by flow cytometry have also been used to isolate CTCs. The DEPArray uses DEP to transfer single cells (viable or fixed) to single wells for downstream processing. However, selection and detection of target cells is based on immunofluorescence staining [42]. The DEPArray shows the highest grade of automation compared to semi-automated micromanipulators like the CellCelector (Automated Lab Solutions), while flow cytometry is a standard procedure in many laboratories for the detection and isolation of CTCs. It is the fastest method with regard to sample processing and readout and provides data on cell count, cell size and marker expression [43]. One disadvantage of immunofluorescence-based systems is their dependency on CTC pre-enrichment. Therefore, many users combine different methods to characterize CTCs, e.g. CellSearch-Flow sorting [43], CellSearch-CellCelector [44] or CellSearch-DEPArray [42].

Different principles of rare cell analysis are summarized in Table 1.

**Table 1:** Enrichment and enumeration based on physical or biological properties of rare cells.

	Physical properties				Biological properties	
	Size/Density/Deformability/Charge				Protein expression/sectrion	
	Centrifugation	Filtration	Microfluidics	Electric. Charge	Immuno-magentics	Immuno-fluorescence
Depletion of Erythrocytes	●	●	●	●	●	
Depletion of Leukocytes		●	●	●	●	
Label-free/marker independent	●	●	●	●		
Enrichment of CTCs		●	●	●	●	●
Harvesting of CTC enriched cell suspension			●	●	●	○
Isolation of rare cells		○			○	●
Direct quantification of rare cells		○	○		○	●
Indirect quantification of rare cells	●	●	●	●	●	●
Example technologies	Ficoll gradient	VyCAP, ISET	Parsortix, Vortex, CTCiChip	Apo-Stream	CellSearch, AdnaGen, Isoflux	Flow Cytometry, Micro-manipulation, DEParray

● Yes.

○ Only for some technologies.

Table 1 focuses on “stand-alone” methods (according to manufacturers' specifications) and does not include possible workflow combinations with other technologies. Downstream analysis technologies can be divided into two groups, based on physical properties and based on biological properties of the cells. “Depletion of erythrocytes” means that methods are able to remove erythrocytes effectively; the same is true for “depletion of leukocytes”. “Label-free” or “marker independent” indicates, if a certain method enriches CTCs independent of marker expression (e.g. EpCAM). “Direct quantification” indicates that a technology is capable of enumeration of CTCs without the use of additional devices. For example, CellSearch performs direct CTC quantification while ISET depends on fluorescence staining and microscopy and AdnaGen uses RT-qPCR to quantify CTC marker expression. If the technologies can be implemented in workflows combining other methods to detect, enumerate and characterize rare cells, this is indicated by “Yes”. A detailed overview of CTC enrichment and isolation technologies has been published by [45].

The combination of different methods will most likely become the standard for single rare cell characterization in oncology. It will be important to optimize such workflows to generate robust, reproducible and cost-efficient workflows, which can also be used in the clinic.

### 2.3 Circulating Cell-Free DNA

Circulating cell-free DNA (ccfDNA) can be isolated from different body fluids, including blood. A passive release by dying cells or active release by secretion are under discussion as sources for ccfDNA [46]. In general, the amount of circulating tumor DNA (ctDNA) increases with tumor burden sums up to 1% of total ccfDNA in early-stage disease and up to 40% in late-stage disease [47] and ctDNA allows detection of a relapse relative early [48, 49]. ctDNA is a valuable biomarker, which is already used for treatment response monitoring or the early detection of relapse [7, 50]. Furthermore, the analysis of ctDNA from patients has an impact on therapy decision (e.g. mut EGFR) [51]. In addition to improving therapy selection, the analysis of ctDNA can be used to monitor the success of a given therapy. For example, Murtaza and colleagues were able to monitor early signs of secondary drug resistance when monitoring cancer patients over a time period of 2 years [52]. The results imply clonal evolution during disease progression due to therapy-induced selective pressure. Finally, with the FDA approved Cobas EGFR Mutation Test v2 (cobas, Roche Diagnostic US, Indianapolis, IN, USA) ctDNA analysis reached the milestone to be used as a companion diagnostic (CDx). This test is used as a CDx for non-small cell lung cancer therapy with Erlotinib.

The size of ctDNA fragements is on average around 160 base pairs (bp). However, there is still an ongoing discussion in the field, whether ctDNA fragments smaller than 100 bp carry relevant information as well [53]. Widely accepted, however, is the fact, that ctDNAs occur fragmented and associated with nucleosomes [54, 55]. Due to the relatively short length of ctDNA fragments and a possibly unequal distribution of released genome fractions, chromosomal aberrations can hardly be detected and even CNVs are difficult to analyze. This has to be considered for other oncogenic alterations, such as chromosomal rearrangements that affect the *BRCA1* and *BRCA2* genes. Similar to CTCs and their low abundance in the blood, ctDNA concentration is low and therefore sensitive downstream analyses are required. Highly specific and sensitive technologies are needed since allelic frequencies as low as 0.01%, representing only small tumor subclones, might be clinically meaningful e.g. for treatment resistance. Optimization of SOPs, benchmarking of different assays and their implementation in analytical sites also require robust reference material and standards for technology assessment. For example, reference material such as synthetic plasma is commercially available (e.g. SeraCare Life Sciences, USA or Horizon Discovery Ltd., UK).

In most cases ctDNA is used to screen to point mutations in a targeted approach to – for example - detect known druggable mutations with potential impact on therapy decision. Emerging technologies for targeted approaches with high sensitivity are digital (droplet) PCR and BEAMing (beads, emulsion, amplification, and magnetics). Next generation sequencing (NGS) is already of great value for ctDNA analysis and liquid biopsy (including mRNA and miRNA sequencing) [[55], [56], [57]]. Recently, a 70-gene panel by Foundation Medicine, the FoundationACT® assay, was granted breakthrough device designation by the FDA (http://investors.foundationmedicine.com/news-releases/news-release-details/foundation-medicines-new-liquid-biopsy-assay-granted), potentially making it the first liquid biopsy NGS panel to achieve regulatory approval. NGS is capable of multi-gene analysis including targeted as well as untargeted detection of point mutations. However, the quality of library construction is of high importance, sensitivity is challenging and bioinformatics evaluation is still labor-intensive and has to be harmonized to generate comparable results [51]. Probably best reflecting the need for standards and benchmarking of different NGS panels is a recent comparative study using NGS panels by two different providers that revealed significant discordance in the detection of genomic alterations in overlapping content of both panels [58]. Big efforts are made to improve sensitivity, specificity and to establish an analysis pipeline for proper variant calling, annotation and filter strategies (reviewed in [56]) and development of technologies such as CAPP-Seq may pave the way for routine detection of low allelic frequency mutations, e.g. for detection of minimal residual disease [59, 60]. CAPP-Seq technology has recently been implemented in the AVENIO ctDNA NGS liquid biopsy assays by Roche, demonstrating the rapid commercial adaption of research in the field. Commercial NGS panel providers increasingly try to enter the field of immune oncology with the aim to position their assays for assessing Tumor Mutational Burden (TMB). Yet, to date, there is no agreement on panel size or content nor on the size of genomic regions to be covered to derive actionable information for a recommendation to start or continue immune checkpoint inhibitor treatment [61].

Another approach is the determination of CNVs, which is still challenging. However, also here few investigators showed successful CNV determination: recently Molparia and colleagues presented the possibility to screen for copy number variations (CNVs) [62, 63]. This allows the use of ctDNA as a (cancer) biomarker not only for the detection of point mutations, but also larger chromosomal aberrations. Detection of epigenetic changes in ctDNA is an additional field of research that receives growing attention [[64], [65], [66]].

**Fig. 1:**
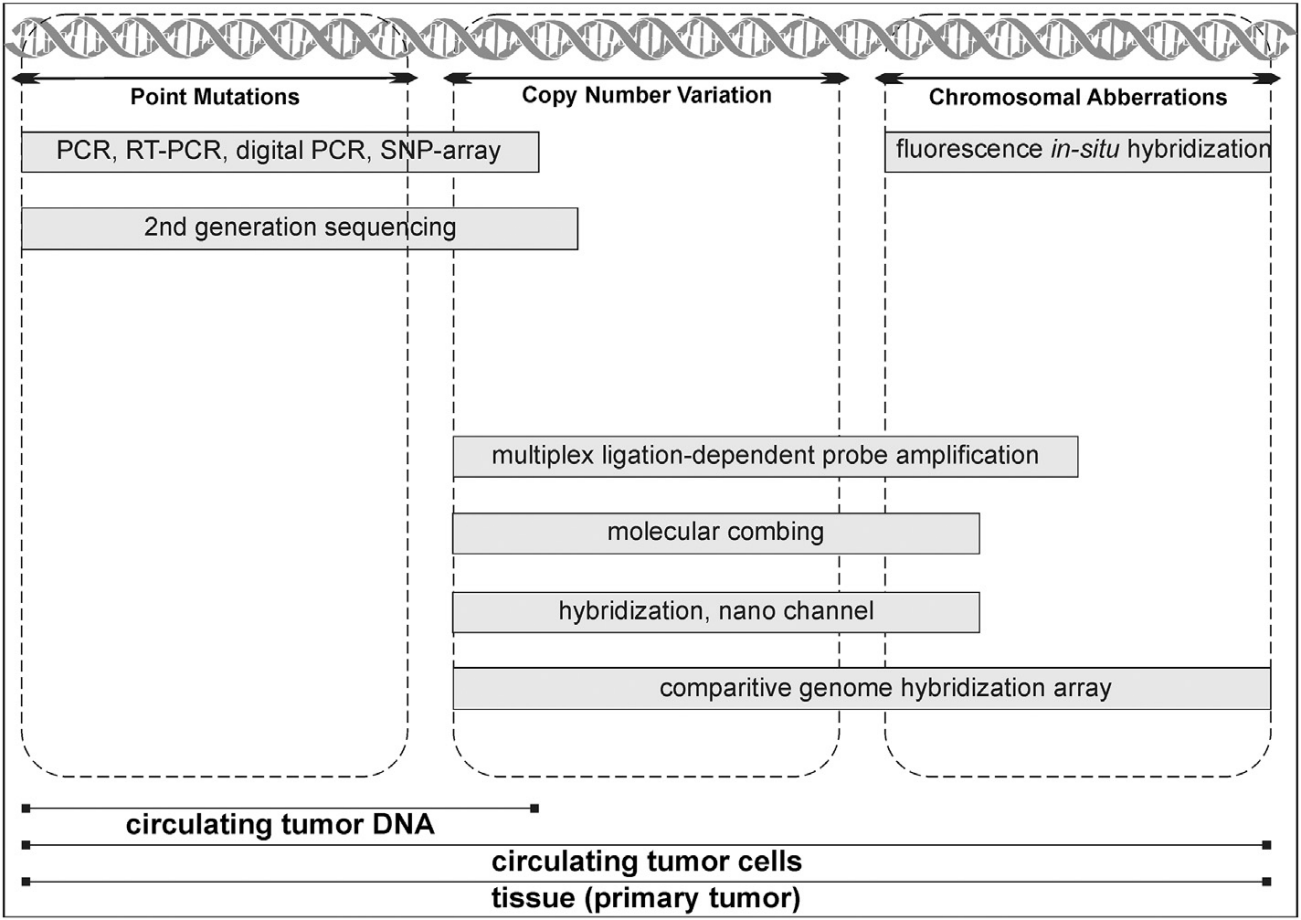
Commercialized methods for analysis of genetic alterations, categorized by the “size” of the genetic alteration. Not all technologies shown here are also currently applied for liquid biopsy or do not have the potential to be applied for it.

Fig. 1 provides an overview of methods used in research or routine diagnostics. The methods are categorized based on the type of genetic alteration (point mutation, CNV or chromosomal aberration). Moreover, it illustrates which methods are currently applied for ctDNA analysis, CTC analysis or routine tissue analysis.

## 3 Outlook

The use of liquid biopsy is a promising concept to comprehensively characterize a patients (malignant) disease in a minimally invasive way. Therefore, it might be used improve early diagnosis and prognosis as well as disease and/or therapy monitoring. Nowadays, liquid biopsy is becoming more and more included in daily clinical practice. However, so far only a few methods, assays or systems have received regulatory approval. An important issue to be further addressed in the future are limitations for parallel detection and quantification of different analytes (ctDNA, CTC, RNA) from a limited volume of blood which are introduced by pre-analyical procedures. In case of ctDNA and CTCs, the low frequency of target mutations found in a cohort of patients requires a cost-effective (pre-) profiling strategy resulting in (pre-) selection of patients. Finally, reliable data evaluation and data interpretation are still challenging. Easy to use bioinformatics tools need to be developed, standardized and validated in comprehensive studies with defined reference material, to result in clinically useful results and a treatment decision.

## Acknowledgement

The authors participate in the Innovative Medicines Initiative consortium CANCER-ID. CANCER-ID is supported by the Innovative Medicines Initiative (IMI) Joint Undertaking under Grant Agreement n° 115,749, resources of which are composed of financial contribution from the European Union's Seventh Framework Programme (FP7/2007–2013) and EFPIA companies' in kind contributions.

## Conflict of Interest

The authors are full-time employees of Bayer AG, Germany. T.K. and T.S. are shareholders of Bayer AG.

## References

[1] Speicher M.R., Pantel K. (2014). Tumor signatures in the blood. Nat Biotechnol.

[2] Sholl L.M. (2016). Liquid biopsy in lung cancer: a perspective from members of the pulmonary pathology society. Arch Pathol Lab Med.

[3] Bedard P.L. (2013). Tumour heterogeneity in the clinic. Nature.

[4] Abbosh C. (2017). Phylogenetic ctDNA analysis depicts early-stage lung cancer evolution. Nature.

[5] Gerlinger M. (2012). Intratumor heterogeneity and branched evolution revealed by multiregion sequencing. N Engl J Med.

[6] Trojan J. (2017). Clinical application of liquid biopsy in targeted therapy of metastatic colorectal cancer. Case Rep Oncol Med.

[7] Schmiegel W. (2017). Blood-based detection of RAS mutations to guide anti-EGFR therapy in colorectal cancer patients: concordance of results from circulating tumor DNA and tissue-based RAS testing. Mol Oncol.

[8] Hawkins R. (2012). Managing the pre- and post-analytical phases of the total testing process. Ann Lab Med.

[9] Plebani M. (2010). The detection and prevention of errors in laboratory medicine. Ann Clin Biochem.

[10] Green S.F. (2013). The cost of poor blood specimen quality and errors in preanalytical processes. Clin Biochem.

[11] Ball J.R., Balogh E. (2016). Improving diagnosis in health care: highlights of a report from the national academies of sciences, engineering, and medicine. Ann Intern Med.

[12] van Ginkel J.H. (2017). Preanalytical blood sample workup for cell-free DNA analysis using Droplet Digital PCR for future molecular cancer diagnostics. Cancer Med.

[13] Page K. (2013). Influence of plasma processing on recovery and analysis of circulating nucleic acids. PLoS One.

[14] Lin C.C. (2015). Emerging platforms using liquid biopsy to detect EGFR mutations in lung cancer. Expert Rev Mol Diagn.

[15] FDA, FDA. Re: K031588 evaluation of automatic class III designation. Cell search epithelial cell kit/cell spotter analyzer.

[16] Chen S. (2017). Microfluidic assay of circulating endothelial cells in coronary artery disease patients with angina pectoris. PLoS One.

[17] Torres C. (2017). Soluble endothelial cell molecules and circulating endothelial cells in patients with venous thromboembolism. Blood Coagul Fibrinolysis.

[18] Zhou F. (2017). Circulating endothelial cells and their subsets: novel biomarkers for cancer. Biomark Med.

[19] Alix-Panabieres C., Pantel K. (2013). Circulating tumor cells: liquid biopsy of cancer. Clin Chem.

[20] Nguyen D.X., Bos P.D., Massague J. (2009). Metastasis: from dissemination to organ-specific colonization. Nat Rev Cancer.

[21] Cristofanilli M. (2004). Circulating tumor cells, disease progression, and survival in metastatic breast cancer. N Engl J Med.

[22] de Bono J.S. (2008). Circulating tumor cells predict survival benefit from treatment in metastatic castration-resistant prostate cancer. Clin Cancer Res.

[23] Giuliano M. (2014). Circulating tumor cells as early predictors of metastatic spread in breast cancer patients with limited metastatic dissemination. Breast Cancer Res.

[24] Stoecklein N.H. (2016). Challenges for CTC-based liquid biopsies: low CTC frequency and diagnostic leukapheresis as a potential solution. Expert Rev Mol Diagn.

[25] Coumans F.A. (2013). Filter characteristics influencing circulating tumor cell enrichment from whole blood. PLoS One.

[26] Shaw Bagnall J. (2015). Deformability of tumor cells versus. Blood Cells Sci Rep.

[27] Lindsay C.R. (2017). A prospective examination of circulating tumor cell profiles in non-small-cell lung cancer molecular subgroups. Ann Oncol.

[28] Ramirez A.B. (2017). RareCyte(R) CTC analysis step 1: AccuCyte(R) sample preparation for the comprehensive recovery of nucleated cells from whole blood. Methods Mol Biol.

[29] de Wit S. (2015). The detection of EpCAM(+) and EpCAM(−) circulating tumor cells. Sci Rep.

[30] Tibbe, A.G.J., C.J.M. Van Rijn, and L.W.M.M. Terstappen, Microsieve Diagnostic Device In The Isolation and Analysis of Single Cells. 2017, Google patents.

[31] Vona G. (2000). Isolation by size of epithelial tumor cells: a new method for the immunomorphological and molecular characterization of circulating tumor cells. Am J Pathol.

[32] Krebs M.G. (2012). Analysis of circulating tumor cells in patients with non-small cell lung cancer using epithelial marker-dependent and -independent approaches. J Thorac Oncol.

[33] Farace F. (2011). A direct comparison of CellSearch and ISET for circulating tumour-cell detection in patients with metastatic carcinomas. Br J Cancer.

[34] Abali F. (2017). Isolation of single cells for protein therapeutics using microwell selection and Surface Plasmon Resonance imaging. Anal Biochem.

[35] Hvichia G., Counts D., Evans G. (2011). Methods and apparatus for segregation of particles.

[36] Chudziak J. (2016). Clinical evaluation of a novel microfluidic device for epitope-independent enrichment of circulating tumour cells in patients with small cell lung cancer. Analyst.

[37] Khoo B.L. (2014). Clinical validation of an ultra high-throughput spiral microfluidics for the detection and enrichment of viable circulating tumor cells. PLoS One.

[38] Sollier E. (2014). Size-selective collection of circulating tumor cells using Vortex technology. Lab Chip.

[39] Che J. (2017). Biophysical isolation and identification of circulating tumor cells. Lab Chip.

[40] Ozkumur E. (2013). Inertial focusing for tumor antigen-dependent and -independent sorting of rare circulating tumor cells. Sci Transl Med.

[41] Jaeger B.A.S. (2017). The HER2 phenotype of circulating tumor cells in HER2-positive early breast cancer: a translational research project of a prospective randomized phase III trial. PLoS One.

[42] Fabbri F. (2013). Detection and recovery of circulating colon cancer cells using a dielectrophoresis-based device: KRAS mutation status in pure CTCs. Cancer Lett.

[43] Neves R.P. (2014). Genomic high-resolution profiling of single CKpos/CD45neg flow-sorting purified circulating tumor cells from patients with metastatic breast cancer. Clin Chem.

[44] Neumann M.H. (2017). Isolation and characterization of circulating tumor cells using a novel workflow combining the CellSearch(R) system and the CellCelector. Biotechnol Prog.

[45] Lampignano R. (2017). Enrichment, isolation and molecular characterization of EpCAM-negative circulating tumor cells. Adv Exp Med Biol.

[46] Stroun M. (2000). The origin and mechanism of circulating DNA. Ann N Y Acad Sci.

[47] Bettegowda C. (2014). Detection of circulating tumor DNA in early- and late-stage human malignancies. Sci Transl Med.

[48] Garcia-Murillas I. (2015). Mutation tracking in circulating tumor DNA predicts relapse in early breast cancer. Sci Transl Med.

[49] Tie J. (2016). Circulating tumor DNA analysis detects minimal residual disease and predicts recurrence in patients with stage II colon cancer. Sci Transl Med.

[50] Siravegna G., Bardelli A. (2014). Genotyping cell-free tumor DNA in the blood to detect residual disease and drug resistance. Genome Biol.

[51] Ulz P. (2017). Patient monitoring through liquid biopsies using circulating tumor DNA. Int J Cancer.

[52] Murtaza M. (2013). Non-invasive analysis of acquired resistance to cancer therapy by sequencing of plasma DNA. Nature.

[53] Mouliere F. (2011). High fragmentation characterizes tumour-derived circulating DNA. PLoS One.

[54] Underhill H.R. (2016). Fragment length of circulating tumor DNA. PLoS Genet.

[55] Ulz P. (2016). Inferring expressed genes by whole-genome sequencing of plasma DNA. Nat Genet.

[56] Perakis S. (2017). Advances in circulating tumor DNA analysis. Adv Clin Chem.

[57] Fernandez-Cuesta L. (2016). Identification of circulating tumor DNA for the early detection of small-cell lung cancer. EBioMedicine.

[58] Torga G., Pienta K.J. (2017). Patient-paired sample congruence between 2 commercial liquid biopsy tests. JAMA Oncol.

[59] Newman A.M. (2014). An ultrasensitive method for quantitating circulating tumor DNA with broad patient coverage. Nat Med.

[60] Chaudhuri A.A. (2017). Early detection of molecular residual disease in localized lung cancer by circulating tumor DNA profiling. Cancer Discov.

[61] Khagi Y. (2017). Hypermutated circulating tumor DNA: correlation with response to checkpoint inhibitor-based immunotherapy. Clin Cancer Res.

[62] Molparia B., Nichani E., Torkamani A. (2017). Assessment of circulating copy number variant detection for cancer screening. PLoS One.

[63] Shaw J.A. (2012). Genomic analysis of circulating cell-free DNA infers breast cancer dormancy. Genome Res.

[64] Pisanic T.R. (2015). DREAMing: a simple and ultrasensitive method for assessing intratumor epigenetic heterogeneity directly from liquid biopsies. Nucleic Acids Res.

[65] Parisi C. (2016). Development and validation of a multiplex methylation specific PCR-coupled liquid bead array for liquid biopsy analysis. Clin Chim Acta.

[66] Nian J. (2017). Diagnostic accuracy of methylated SEPT9 for blood-based colorectal cancer detection: a systematic review and meta-analysis. Clin Transl Gastroenterol.

